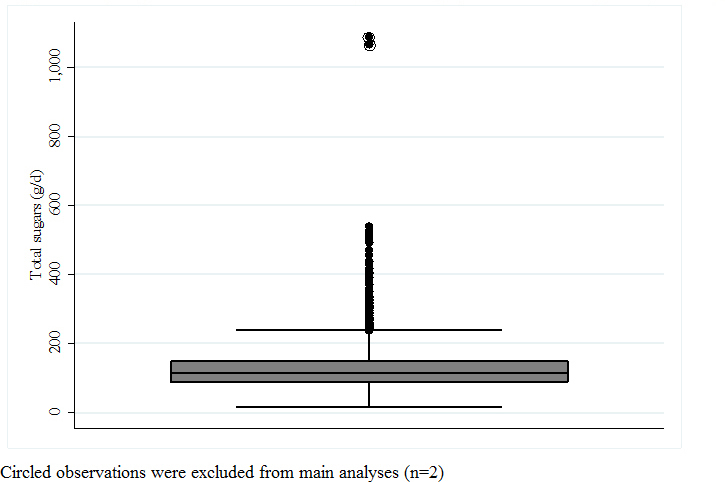# Intakes and sources of dietary sugars and their association with metabolic and inflammatory markers

**DOI:** 10.1016/j.clnu.2017.05.030

**Published:** 2018-08

**Authors:** Laura O'Connor, Fumiaki Imamura, Soren Brage, Simon J. Griffin, Nicholas J. Wareham, Nita G. Forouhi

**Affiliations:** a MRC Epidemiology Unit, University of Cambridge School of Clinical Medicine, Institute of Metabolic Science, Cambridge Biomedical Campus, Cambridge, UK; b Department of Health Professions, Faculty of Health, Psychology and Social Care, Manchester Metropolitan University, Manchester, UK; c Primary Care Unit, Department of Public Health and Primary Care, University of Cambridge School of Clinical Medicine, Institute of Public Health, Cambridge Biomedical Campus, Cambridge, UK

**Keywords:** Sugars, Free sugar, Metabolic, Inflammation, Glycaemia, CRP, C-reactive protein, HbA_1c_, glycated haemoglobin, FFQ, food frequency questionnaire, HOMA-IR, homeostasis model assessment of insulin resistance, EPIC, The European Prospective Investigation into Cancer and Nutrition, NSP, non-starch polysaccharides, %TEI, percent total energy intake, PAEE, physical activity energy expenditure, WHO, World Health Organisation

## Abstract

**Background & aims:**

Associations of dietary sugars with metabolic and inflammatory markers may vary according to the source of the sugars. The aim of this study was to examine the association of dietary sugars from different sources [beverages (liquids), foods (solids), extrinsic (free) or intrinsic (non-free)] with metabolic and inflammatory markers.

**Methods:**

Population-based cross-sectional study of adults in the East of England (n = 9678). Sugar intakes were estimated using food frequency questionnaires. Fasting glycated haemoglobin, glucose, insulin, and C-Reactive Protein (CRP) were measured and indices of metabolic risk were derived (homeostatic model of insulin resistance, HOMA-IR and metabolic risk z-score).

**Results:**

In multiple linear regression analyses adjusted for potential confounders including BMI and TEI, sugars from liquids were positively associated with ln-CRP [b-coefficient (95%CI), 0.14 (0.05,0.22) per 10%TEI] and metabolic risk z-score [0.13 (0.07,0.18)]. Free sugars were positively associated with ln-HOMA-IR [0.05 (0.03,0.08)] and metabolic risk z-score [0.09 (0.06,0.12)]. Sugars from solids were not associated with any outcome. Among major dietary contributors to intakes (g/d), sugars in fruit, vegetables, dairy products/egg dishes, cakes/biscuits/confectionary and squash/juice drinks were not associated, but sugar added to tea, coffee, cereal was significantly positively associated with all outcomes. Sugars in 100% juice [0.16 (0.06,0.25) per 10%TEI] and other non-alcoholic beverages [0.13 (0.03,0.23)] were positively associated with metabolic risk z-score.

**Conclusion:**

Higher intakes of sugars from non-alcoholic beverages and sugar added to tea, coffee, cereal were associated with glycaemia and inflammatory markers. Sugars from solids were not associated, irrespective of whether they were intrinsic or extrinsic. Positive associations of free sugars were largely explained by contribution of beverages to intake.

## 1 Introduction

The role of dietary sugars in the aetiology of cardio-metabolic disease has been long debated [1], [2]. As cardio-metabolic diseases are largely preventable, the identification of modifiable factors that influence the pathogenesis of these diseases is central to combatting their onset. Thus, the association of dietary sugars with cardio-metabolic disease warrants further attention and clarification.

In recent years evidence has accumulated that dietary sugars are associated with increased body-weight, as summarised in a large meta-analysis of 30 randomised controlled trials and 38 cohort studies [3]. It is now widely accepted that dietary sugars promote adverse metabolic outcomes via weight-gain through their contribution to energy intake. There is also emerging evidence that dietary sugars, including sucrose or other mono- and di-saccharides or free sugars intake, are associated with increased blood pressure and serum lipids, independently of body fat [4]. There is thus a suggestion that dietary sugars may also be associated with increased metabolic risk, independently of energy intake and body-weight.

Dietary sugars are a complex component of the diet and their effects on health outcomes are likely to differ depending on the properties of the consumed sugars, including chemical composition (e.g. glucose versus fructose), and the source of the sugars, e.g. from beverage or food sources or, extrinsic or intrinsic cellular location in the food. However, research focus to date has largely been on the health effects associated with intakes of total sugars [5], added sugars [6], individual sugars, in particular fructose [7] and intakes of sugary beverages [8], [9] but evidence for differential association of sugars from different sources with different physical properties is limited.

The aim of this study was to examine the association between intakes of dietary sugars from different sources [beverages (sugars from liquids), food (sugars from solids), extrinsic (free) sugars, intrinsic (non-free) sugars] and metabolic markers including, glycated haemoglobin (HbA_1c_), homeostasis model assessment of insulin resistance (HOMA-IR), C-reactive protein (CRP) and a metabolic risk z-score.

## 2 Materials and methods

### 2.1 Study design and population

The Fenland Study is a population-based observational study. Participants born between 1950 and 1975 were recruited from general practice lists in and around Cambridgeshire, in the East of England, UK. In total, 12,434 participants were enrolled between 2005 and 2015. Exclusion criteria of the Fenland study included pregnancy, physician-diagnosed diabetes, inability to walk unaided, psychosis, or terminal illness. Participants missing any exposure or outcome data (n = 2754) and participants with extremely high intakes of total sugars (n = 2) (Supplementary Fig. 1) were excluded, leaving 9678 participants for inclusion in these analyses. Ethical approval was granted by the Cambridge Local Research Ethics Committee. All participants gave written informed consent.

### 2.2 Dietary assessment

Habitual diet over the previous year was self-reported using a validated 130 item semi-quantitative food frequency questionnaire (FFQ) [10]. Total energy intake, nutrient intake including intake of total sugars, and food group intakes were estimated as previously described [11], using the UK's food compositional tables, McCance and Widdowson's, *The Composition of Foods*. In a validation study, the Spearman correlation coefficient between individual results from 16-day weighed dietary records and FFQs for total sugar (g/d) was 0.51 with 44% of participants classified into the same quartiles of intakes, 51% in adjacent quartiles and 5% into extreme quartiles [12].

Total sugars included monosaccharides and disaccharides from all sources. Intakes of sugars from liquids, sugars from solids, free-sugars and non-free-sugars were estimated post hoc by categorising food sources as follows. Sugars from liquids were estimated as total sugars from beverages which included: teas, coffees, hot-chocolate, malted-milk drinks, alcoholic beverages, fizzy drinks, fruit juice and fruit squash. Sugars from solids were estimated as total sugars from foods. Foods included all foods including semi-solid foods like yoghurt and soups. In primary analyses, milk was excluded from sugars in liquids or solids as it was not discerned using the FFQ whether milk was consumed as a beverage or in food e.g. milk in cereal or composite dishes: the influence of this decision was assessed in sensitivity analysis (see below). Information on table sugar added to tea, coffee, cereal was collected as a separate single question in the FFQ and could not be directly linked to the food or beverage with which it was consumed. As such all table sugar added to tea, coffee, or cereal by the participant was included as sugars from solids.

Free sugars were estimated according to the Scientific Advisory Committee of Nutrition's (SACN's) and the World Health Organisation's (WHO's) definition [5], and were calculated using a combination of published methods [13] as no single method was fully comprehensive. Free sugars included: 100% of total sugar from fruit juice, table sugar, honey, syrups; 100% of sugar in processed foods where the unprocessed product has no naturally occurring sugar e.g. meat; 50% of total sugar in processed foods which also had naturally occurring sugar e.g. refined cereal, baked beans. Sugar in milk was excluded but, sugar in dairy products and milk based beverages was included as: total sugar minus lactose. Sugars in canned, stewed and dried fruit were not included as free sugars as per SACN's and the WHO's definition but, sugar in sweetened versions of these, e.g., fruit canned in syrup was included as sugar in sweetened product minus sugar in unsweetened product. Non-free sugars were estimated as total sugars minus free sugars.

### 2.3 Clinical and biochemical measurements

Height, weight, waist circumference and blood pressure were measured and BMI was calculated using standardised methods (Supplementary text). Fasting venous blood samples were taken and were followed by a standard 75 g oral glucose tolerance test with further samples taken at 120 min. Plasma glucose, triglycerides (TG) HDL cholesterol, insulin, HbA_1c_ and CRP levels were measured using standardised techniques (Supplementary text). HOMA-IR was calculated to evaluate insulin resistance (fasting insulin (μU/ml) × fasting plasma glucose (mmol/L)/22.5).

We constructed a standardised continuous variable for the metabolic risk, broadly based on the definition proposed by the WHO and described previously in detail [14], [15]. The variable was derived by summing the z-scores of continuous indices of anthropometry (waist circumference), blood pressure (systolic blood pressure and diastolic blood pressure), glycaemia (2-h plasma glucose), insulin (fasting insulin), lipids (inverted fasting HDL cholesterol) and triglycerides. The summed score was further scaled to have one standard deviation and hereafter referred to as the metabolic risk z-score.

### 2.4 Covariates

Self-reported demographic, lifestyle and health variables were collected using a questionnaire. These included age, sex, marital status (single, married, widowed/separated/divorced), age at completion of full-time education, income level (<£20,000, £20,000–£40,000, >£40,000), social class (routine and manual occupations, intermediate occupations and higher managerial, administrative and professional occupations), smoking status (never, former, current), alcohol intake (units/week), and being on a weight-loss diet (yes, no). Information on test site location (Ely, Wisbech, Cambridge), self-reported hypertension or hyperlipidaemia and the use of anti-hypertensive and lipid lowering medication were also recorded. Physical activity was objectively assessed over 6 days using a combined heart rate and movement sensor (Actiheart, CamNTech, Cambridge, UK), with individual calibration of heart rate performed using a treadmill test. Data from free-living was pre-processed and modelled using a branched equation framework to estimate intensity time-series, which were summarised over time as daily Physical Activity Energy Expenditure (PAEE) (kJ/kg/d) [16]. Plasma vitamin C is an objective marker of fruit and vegetable intake [17] and was used here as a proxy of dietary quality. For plasma vitamin C measurement, blood samples were taken into heparin tubes, centrifuged, aliquoted, stabilised with metaphosphoric acid, and stored (detail in Supplementary text). Plasma vitamin C concentration was measured by fluorometric assay within 2 months.

Participants with missing covariate data were retained for analysis; missing data in categorical variables were coded as missing. Where objectively measured physical activity measurement was not available (n = 102), self-reported data from a validated questionnaire, the Recent Physical Activity Questionnaire (RPAQ) [18] were used. Where age at completion of full-time education was not available (n = 270), we used the mean of a matched sample based on age, sex, household income level and social class.

### 2.5 Statistics

Population characteristics of the total study population (n = 12,434) were compared with those excluded from the analysis (n = 2756). Intakes of sugars from liquids, sugars from solids, free sugars and non-free sugars were expressed as % contribution to total energy intake (%TEI). Each sugar exposure (%TEI) was split by quintile into consumption categories. The population characteristics [mean ± standard deviation (SD), median (inter-quartile range) or percent] of the highest consumption category were compared with those of the lowest consumption category for each sugar intake exposure. *P*-values for trend for population characteristics were estimated across quintiles using ANOVA or chi-squared for independence.

We used multiple linear regression to assess the categorical (quintile) and continuous (per 10%TEI) associations of each sugar intake exposure with HbA_1c_, HOMA-IR, CRP and the metabolic risk z-score. HOMA-IR and CRP were natural-log (ln) transformed. *P-*values for trend across quintiles were estimated by including the median value of each quintile and modelling as a continuous variable. Model 1 was adjusted for age, sex, years of education, income level, social class, smoking status, alcohol consumption, test site, PAEE, clinical history (self-reported medication use for hypertension or hyperlipidaemia), self-reported weight-loss diet (yes/no), fibre (non-starch polysaccharides, NSP) intake and plasma vitamin C measurement as proxies of dietary quality, non-sugar containing beverage intake (tea, coffee and artificially sweetened beverages) and low-nutrient energy-dense food intake (buns, cakes, puddings, biscuits, pastries, chocolates and non-chocolate confectionary and ice-cream). Model 2 was additionally adjusted for BMI, energy intake and mutually adjusted for intakes of other sugars.

A number of sensitivity analyses and tests for interaction were pre-specified. Sensitivity analyses included: [1] using energy partition and residual methods [19] in place of the above-mentioned nutrient density (%TEI) approach, to characterise the influence of TEI; [2] including total sugars from milk as sugars from liquids; [3] including sugar added to tea, coffee, cereal as sugars from liquids rather than sugars from solids; [4] restricting analyses of HbA_1c_ to participants with levels of HbA_1c_ < 6.5% (thus excluding those potentially with undiagnosed T2D). Possible interactions with age (continuous), sex (dichotomous), BMI (continuous), PAEE (continuous) and smoking status (dichotomous) were examined by including each interaction term with a sugar exposure in Model 2. Where interactions were significant (p < 0.05), stratified analyses were conducted.

Macronutrient substitution models were also constructed to examine the effect of replacing sugars with each of polyols, oligo-saccharides and starch, total fat, and total protein (using Model 2) [19]. We assessed the association of sugars from liquids, sugars from solids, free sugars and non-free sugars (per 10%TEI) with the individual components of the metabolic risk z-score using Model 2. Fasting insulin and triglycerides were natural log (ln) transformed. We identified major food and beverage contributors to intake of each of sugars from liquids, sugars from solids, free sugars and non-free sugar as those that contributed >10% of intake (g/d) of each sugar exposure. We then examined the associations of sugars from each of the major foods and beverage contributors with HbA_1c_, ln HOMA-IR, ln CRP and the metabolic risk z-score, using Model 2 which was mutually adjusted for sugars from all other sources.

Intakes of dietary sugars and their major food and beverage contributors were reported by BMI status (normal weight (<25 kg/m^2^), overweight (25–30 kg/m^2^), obese (>30 kg/m^2^)) to inform our interpretation of results given the possibility of reverse causality in cross-sectional analyses.

The analyses were performed using Stata (version 13; Stata Corp, TX, USA). Statistical significance was set at p ≤ 0.003 to account for multiple testing (4 exposures × 4 outcomes).

## 3 Results

### 3.1 Intakes and sources of dietary sugars

**Fig. 1:**
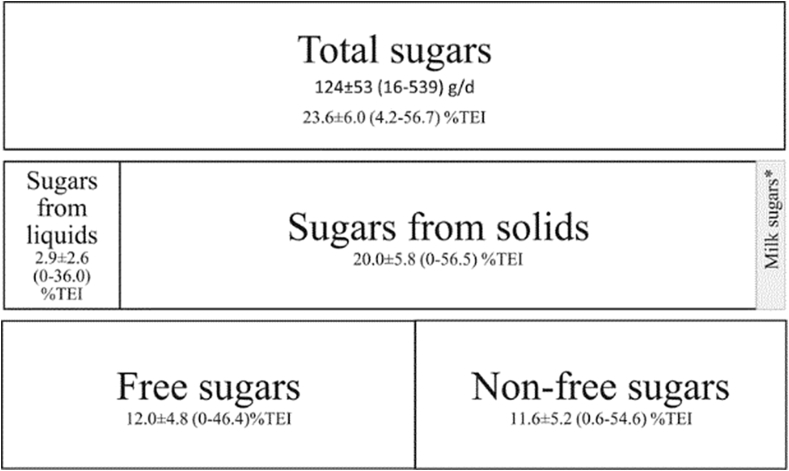
Contribution of dietary sources of sugars to intakes of total sugars (%TEI); The Fenland Study, UK (n = 9678). Abbreviations: Percent Total Energy Intake, %TEI. Values are: mean ± SD (range). *Milk sugars were not included as an exposure in these analyses as the food frequency questionnaire did not enable determining if milk was consumed as a beverage or in a composite food.

The mean ± SD intake of total sugars in this study population was 124 ± 53 g/d, representing 23.6 ± 6.0%TEI (Fig. 1). The majority (85%) of total sugars were consumed as sugars from solids, 20.0 ± 5.8%TEI. Sugars from liquids contributed only a small amount (12%) to intake of total sugars, 2.9 ± 2.6%TEI, and there were 10% non-consumers in this population. All sugars from liquids and 45% of sugars from solids were consumed as free sugars. Free sugars and non-free sugars contributed similarly (50% each) to intake of total sugars, 12.0 ± 4.8 and 11.6 ± 5.2 %TEI, respectively.

### 3.2 Population characteristics

Population characteristics of the total study population included for analyses (n = 9678) and those excluded due to missing exposure or outcome data (n = 2756) were broadly similar (Supplementary Table 1).

Table 1 shows the population characteristics by consumption category (quintile) of each of sugars from liquids, sugars from solids, free sugars and non-free sugars. Those with higher intakes of sugars from liquids were more likely to be younger, to be men, to be single, to have a higher BMI and to consume more alcohol. They were also more likely to have a higher education level, household income and social class, to smoke less and to have higher PAEE. Participants with higher intakes of free sugar were more likely to be younger and to be men, with lower education, household income and social class and to smoke more, but also to have higher PAEE and consume less alcohol. Socio-demographic characteristics by consumption category of sugars from solids and non-free sugars were similar to each other and were largely in the opposite direction to that for free sugars.

**Table 1:** Population characteristics by consumption category (quintile) of each of sugars from liquids, sugars from solids, free sugars and non-free sugars (%TEI); The Fenland Study, UK (n = 9678).

	Sugars from liquids		Sugars from solids		Free sugars		Non-free sugars	
	Q1	Q5	Q1	Q5	Q1	Q5	Q1	Q5
Age (year)	48.5 ± 7.2	46.1 ± 7.5*	47.3 ± 7.5	48.2 ± 7.3*	48.8 ± 7.3	46.7 ± 7.4*	46.9 ± 7.4	48.8 ± 7.3*
Sex, men (%)	38.1	48.6*	56.6	34.3*	39.1	52.28*	64.9	30.8*
Marital status, married (%)	60.3	56.4*	57.9	55.7	58.8	55.5	57.5	56.4
Age at completion of full-time education (year)	18.5 ± 4.1	18.6 ± 3.7*	19.1 ± 4.4	18.6 ± 4.2	19.0 ± 4.5	18.2 ± 3.6*	18.4 ± 4.0	18.8 ± 4.3*
Household income level, >£40,000 per year (%)	41.1	48.8*	51.2	45.0	49.0	42.1*	43.3	49.2*
Social class, managerial & professional occupations (%)	48.8	52.8*	55.1	53.1	56.0	47.0*	46.2	56.6*
Smoking status, current (%)	15.2	13.4*	18.9	12.2*	13.3	18.1*	23.3	6.8*
Alcohol consumption (units/week)	1.6 (0.0,5.2)	5.4 (1.3,11.9)*	8.5 (2.7,24.5)	1.8 (0,5.6)*	4.8 (1.1,9.6)	4.1 (0.7,8.4)*	6.4 (1.4,14.7)	2.7 (0.7,7.5)*
BMI (kg/m2)	26.9 ± 5.1	27.1 ± 4.8*	27.4 ± 4.9	26.6 ± 5.0*	27.0 ± 5.0	27.0 ± 4.9	27.5 ± 5.0	26.7 ± 5.0*
PAEE (kJ/kg/d)	52.3 ± 22.3	55.9 ± 23.7*	54.1 ± 22.8	54.7 ± 24.1	52.1 ± 22.1	57.3 ± 24.6*	56.1 ± 24.0	53.7 ± 22.7*
*Dietary exposures*								
Energy (kJ/d)	7816 ± 2871	8284 ± 2677*	8107 ± 2784	8037 ± 2881*	7217 ± 2522	9418 ± 3392*	9396 ± 3480	7189 ± 2317*
Total fat (%TEI)	34.7 ± 6.7	31.4 ± 5.5*	36.2 ± 6.4	29.5 ± 5.38	33.6 ± 7.0	33.1 ± 5.5*	37.4 ± 5.6	28.5 ± 5.0*
Total protein (%TEI)	18.9 ± 4.0	17.1 ± 3.3*	18.7 ± 3.9	17.4 ± 3.7*	20.5 ± 3.9	15.5 ± 2.9*	16.7 ± 3.8	19.0 ± 3.6*
Total carbohydrates (%TEI)	48.0 ± 7.6	50.2 ± 7.3*	41.6 ± 6.9	54.8 ± 5.5*	44.9 ± 8.3	52.3 ± 5.8*	44.2 ± 7.3	53.5 ± 6.2*
Total sugars (%TEI)	22.2 ± 6.4	26.6 ± 6.1*	16.6 ± 3.9	31.5 ± 4.7*	19.7 ± 6.5	28.7 ± 5.2*	19.4 ± 5.7	29.7 ± 5.3*
Sugars from liquids (%TEI)	0.5 ± 0.3	7.0 ± 2.9*	3.5 ± 3.2	2.5 ± 2.5*	1.5 ± 1.2	4.9 ± 4.0*	3.2 ± 3.1	2.7 ± 2.5*
Sugars from solids (%TEI)	21.7 ± 6.4	19.5 ± 5.6*	13.2 ± 2.4	28.9 ± 4.1*	18.2 ± 6.6	23.8 ± 5.5*	16.1 ± 5.2	26.9 ± 5.0*
Free sugars (%TEI)	9.9 ± 4.7	15.6 ± 4.9*	9.4 ± 4.0	14.3 ± 6.2*	6.2 ± 1.4	19.2 ± 3.8*	13.8 ± 5.7	10.2 ± 4.1*
Non-free sugars (%TEI)	12.3 ± 5.9	10.9 ± 5.0*	7.3 ± 2.4	17.2 ± 6.5*	13.6 ± 6.3	9.8 ± 4.2*	5.6 ± 1.3	19.5 ± 4.4*
Starch (%TEI)	24.7 ± 6.2	22.0 ± 4.9*	24.3 ± 6.5	21.3 ± 4.6*	24.2 ± 6.6	21.7 ± 4.6*	23.8 ± 5.7	22.1 ± 5.1*
Fibre, NSP (g/d)	18.0 ± 7.7	16.8 ± 6.7*	15.3 ± 6.5	20.1 ± 8.4*	18.0 ± 8.2	17.0 ± 6.9*	15.2 ± 6.3	21.0 ± 8.5*
Fruit (g/d)	247 ± 223	247 ± 217	118 ± 88	432 ± 305*	278 ± 246	218 ± 200*	89 ± 74	485 ± 290*
Vegetables (g/d)	270 ± 168	256 ± 132*	241 ± 128	287 ± 180*	292 ± 187	243 ± 130*	224 ± 117	313 ± 190*
Total fish (g/d)	42 ± 38	42 ± 35*	46 ± 42	39 ± 31*	48 ± 41	37 ± 30*	42 ± 40	43 ± 33
Red meat & processed meat (g/d)	53 ± 43	53 ± 37*	68 ± 51	39 ± 31*	55 ± 48	54 ± 36	72 ± 49	37 ± 30*
SSB (g/d)	4 ± 7	130 ± 173*	52 ± 122	35 ± 90*	9 ± 19	110 ± 172*	75 ± 145	27 ± 73*
Fruit juice (g/d)	7 ± 10	116 ± 123*	54 ± 78	53 ± 78*	29 ± 42	82 ± 111*	51 ± 76	58 ± 82*
Plasma vitamin C (μmol/L)	63.9 ± 23.1	70.9 ± 21.1*	63.4 ± 22.1	71.7 ± 21.2*	68.3 ± 21.8	65.8 ± 22.4*	58.7 ± 23.2	75.3 ± 19.2*
*Outcomes*								
HbA1c (%)	5.5 (5.3,5.7)	5.5 (5.3,5.7)*	5.5 (5.3,5.7)	5.5 (5.3,5.7)	5.5 (5.3,5.7)	5.5 (5.3,5.7)	5.5 (5.3,5.8)	5.5 (5.3,5.7)*
HOMA-IR	1.2 (0.8,1.7)	1.2 (0.8,2.0)	1.3 (0.8,2.0)	1.1 (0.7,1.7)*	1.1 (0.8,1.7)	1.3 (0.8,2.0)	1.4 (0.9,2.2)	1.4 (0.9,2.2)*
CRP (mg/L)	1.34 (0.55,3.23)	1.48 (0.62,3.41)	1.57 (0.65,3.55)	1.28 (0.53,3.06)*	1.39 (0.60,3.27)	1.50 (0.63,3.33)	1.65 (0.65,3.55)	1.26 (0.52,3.06)*
Metabolic risk z score	−0.05 ± 1.0	0.08 ± 1.00*	0.16 ± 1.1	−0.12 ± 0.96	−0.06 ± 1.0	0.11 ± 0.97*	0.26 ± 1.1	−0.16 ± 0.98*

* Significant p for trend (<0.05) estimated across all five consumption categories (Q1 through Q5) using ANOVA or chi-square test for independence.

Values are: mean ± SD, median (inter-quartile range) or %.

SSB include soft drinks and fruit squash/juice drink.

Metabolic risk z score: derived by summing the z-scores of the following continuous indices: waist circumference, systolic blood pressure + diastolic blood pressure, 2 h plasma glucose, fasting insulin, inverted fasting HDL cholesterol, and triglycerides.

Abbreviations: C-Reactive Protein, CRP; Glycated Haemoglobin, HbA1c; Homeostasis Model Assessment of Insulin Resistance, HOMA-IR; Non-Starch Polysaccharide, NSP; Percent Total Energy Intake, %TEI; Physical Activity Energy Expenditure, PAEE; Quintiles, Q; Sugar Sweetened beverages, SSB.

Higher intake of each source of sugars was associated with lower total fat and starch (%TEI) and higher total carbohydrates and total sugars (%TEI). Higher intakes of sugars from liquids and free sugars were associated with higher energy intake and lower protein (%TEI), fibre (g/d) and vegetable intake. For intakes of sugars from solids and non-free sugars, these associations with dietary intakes were in the opposite direction.

Notably, there was no difference in fruit intake across the consumption categories of sugars from liquids, although those with higher intakes of sugars from liquids had higher plasma vitamin C measurements. Both fruit intake and plasma vitamin C levels were higher in those with higher intakes of sugars from solids and non-free sugars. Those with higher intakes of free sugars had lower intakes of fruit and lower plasma vitamin C levels.

### 3.3 Metabolic and inflammatory markers

After correction for multiple testing (α = 0.003), intake of sugars from liquids (%TEI) was positively associated with ln HOMA-IR [Q5 versus Q1: β-coefficient (95% confidence intervals, 95%CI), 0.11 (0.07,0.15), p-trend < 0.001], ln CRP [0.21 (0.13,0.28), p-trend < 0.001] and the metabolic risk z-score [0.18 (0.13,0.24), p-trend < 0.001] and not significantly associated with HbA_1c_ [0.00 (−0.01,0.04), p-trend = 0.058] (Model 1, Table 2). Adjustment for BMI, TEI and mutual adjustment for sugars (Model 2) attenuated the magnitude of the associations but they remained significantly positively associated. Higher intakes of free sugars were positively associated with ln HOMA-IR and the metabolic risk z-score [Q5 versus Q1: β-coefficient (95%CI), 0.08 (0.04,0.12), p-trend < 0.001 and 0.13 (0.08,0.17), p-trend < 0.001], Model 2. Intakes of sugars from solids and of non-free sugars were inversely associated with ln HOMA-IR when highest consumers (Q5) were compared with lowest consumers (Q1) (Model 1). The association of sugars from solids was attenuated to the null after adjustment for BMI, TEI and mutual adjustment (Model 2). The association of non-free sugars was attenuated slightly but remained significant after further adjustment [β-coefficient (95%CI),−0.07 (−0.11,−0.03), p-trend = 0.001].

**Table 2:** The association of intakes of sugars from liquids, sugars from solids, free sugars and non-free sugars (%TEI) and metabolic & inflammatory markers using multiple linear regression; The Fenland Study, UK (n = 9678).

		Sugars from liquids						Sugars from solids					
		Q1 n = 1936	Q2 n = 1936	Q3 n = 1935	Q4 n = 1936	Q5 n = 1935	p-trend*	Q1 n = 1936	Q2 n = 1936	Q3 n = 1935	Q4 n = 1936	Q5 n = 1935	p-trend*
Intake range (%TEI)		0–0.9	0.9–1.9	1.9–2.8	2.8–4.3	4.3–36.0		2.4–15.9	16.0–18.9	19.0–21.6	21.6–24.8	24.8–56.5	
HbA1c (%)	Model 1	ref	−0.02 (−0.04,0.01)	−0.02 (−0.04,0.01)	−0.02 (−0.04,0.01)	0.02 (−0.01,0.04)	0.058	ref	−0.03 (−0.06,−0.01)	−0.01 (−0.04,0.02)	−0.04 (−0.06,−0.01)	−0.02 (−0.04,0.01)	0.357
	Model 2	ref	−0.02 (−0.04,0.01)	−0.02 (−0.05,0.01)	−0.02 (−0.05,0.00)	0.00 (−0.02,0.03)	0.393	ref	−0.02 (−0.05,0.00)	0.00 (−0.02,0.03)	−0.02 (−0.05,0.01)	0.01 (−0.02,0.04)	0.445
ln HOMA-IR	Model 1	ref	0.03 (−0.00,0.07)	0.04 (−0.00,0.07)	0.09 (0.05,0.13)**	0.11 (0.07,0.15)**	<0.001	ref	−0.02 (−0.06,0.02)	−0.04 (−0.08,0.00)	−0.08 (−0.12,−0.04)**	−0.08 (−0.12,−0.04)**	<0.001
	Model 2	ref	0.03 (0.00,0.06)	0.02 (−0.01,0.06)	0.07 (0.03,0.10)**	0.06 (0.03,0.10)**	<0.001	ref	0.01 (−0.02,0.04)	0.00 (−0.03,0.04)	−0.02 (−0.05,0.02)	−0.02 (−0.06,0.02)	0.109
ln CRP (mg/L)	Model 1	ref	0.04 (−0.03,0.12)	0.05 (−0.02,0.13)	0.13 (0.06,0.21)**	0.21 (0.13,0.28)**	<0.001	ref	−0.04 (−0.11,0.04)	−0.06 (−0.14,0.02)	−0.03 (−0.10,0.05)	−0.09 (−0.17,−0.01)	0.067
	Model 2	ref	0.04 (−0.03,0.11)	0.04 (−0.03,0.11)	0.10 (0.03,0.17)	0.14 (0.06,0.21)**	<0.001	ref	0.01 (−0.06,0.08)	−0.00 (−0.07,0.07)	0.06 (−0.01,0.14)	0.00 (−0.07,0.08)	0.601
Metabolic risk z score	Model 1	ref	0.00 (−0.05,0.06)	0.06 (0.01,0.12)	0.12 (0.06,0.17)**	0.18 (0.13,0.24)**	<0.001	ref	−0.06 (−0.11,−0.00)	−0.07 (−0.13,−0.02)	−0.07 (−0.13,−0.02)	−0.08 (−0.13,−0.02)	0.014
	Model 2	ref	0.01 (−0.03,0.05)	0.05 (0.01,0.09)	0.08 (0.03,0.12)**	0.11 (0.07,0.15)**	<0.001	ref	−0.01 (−0.05,0.03)	−0.02 (−0.06,0.02)	0.02 (−0.03,0.06)	0.01 (−0.04,0.05)	0.474
		Free sugars						Non-free sugars					
		Q1 n = 1936	Q2 n = 1935	Q3 n = 1936	Q4 n = 1935	Q5 n = 1935	p-trend*	Q1 n = 1936	Q2 n = 1935	Q3 n = 1936	Q4 n = 1935	Q5 n = 1935	p-trend*
Intake range (%TEI)		0.5–8.0	8.0–10.4	10.4–12.6	12.6–15.5	15.5–46.4		0.6–7.3	7.3–9.6	9.6–11.9	11.9–15.2	15.2–54.6	
HbA1c (%)	Model 1	ref	−0.01 (−0.04,0.02)	−0.00 (−0.03,0.02)	0.03 (−0.00,0.05)	0.00 (−0.03,0.03)	0.388	ref	−0.02 (−0.04,0.01)	−0.03 (−0.06,−0.00)	−0.02 (−0.05,0.01)	−0.03 (−0.06,0.00)	0.127
	Model 2	ref	−0.01 (−0.03,0.02)	0.00 (−0.02,0.03)	0.03 (−0.00,0.05)	0.00 (−0.03,0.03)	0.303	ref	−0.01 (−0.04,0.01)	−0.02 (−0.05,0.01)	−0.01 (−0.04,0.02)	−0.01 (−0.05,0.02)	0.616
ln HOMA-IR	Model 1	ref	0.03 (−0.01,0.07)	0.05 (0.01,0.09)	0.05 (0.01,0.09)	0.07 (0.02,0.11)**	0.002	ref	−0.03 (−0.07,0.01)	−0.03 (−0.07,0.01)	−0.07 (−0.11,−0.03)**	−0.09 (−0.13,−0.05)**	<0.001
	Model 2	ref	0.04 (0.01,0.07)	0.07 (0.04,0.10)**	0.06 (0.02,0.09)**	0.08 (0.04,0.12)**	<0.001	ref	−0.02 (−0.06,0.01)	−0.02 (−0.05,0.01)	−0.04 (−0.07,−0.00)	−0.07 (−0.11,−0.03)**	0.001
ln CRP (mg/L)	Model 1	ref	−0.00 (−0.08,0.07)	−0.02 (−0.09,0.06)	0.04 (−0.04,0.11)	0.08 (−0.01,0.16)	0.051	ref	0.00 (−0.07,0.08)	0.01 (−0.07,0.08)	−0.04 (−0.12,0.04)	−0.03 (−0.12,0.06)	0.719
	Model 2	ref	0.01 (−0.06,0.08)	0.01 (−0.06,0.08)	0.05 (−0.03,0.12)	0.10 (0.02,0.18)	0.012	ref	0.01 (−0.06,0.08)	0.03 (−0.05,0.10)	0.01 (−0.06,0.09)	0.00 (−0.08,0.09)	0.999
Metabolic risk z score	Model 1	ref	0.03 (−0.02,0.08)	0.03 (−0.02,0.08)	0.06 (−0.00,0.11)	0.11 (0.04,0.17)**	<0.001	ref	−0.03 (−0.08,0.03)	−0.03 (−0.09,0.02)	−0.07 (−0.13,−0.02)	−0.04 (−0.10,0.02)	0.122
	Model 2	ref	0.05 (0.00,0.09)	0.06 (0.02,0.10)**	0.07 (0.03,0.11)**	0.13 (0.08,0.17)**	<0.001	ref	−0.02 (−0.06,0.02)	−0.02 (−0.06,0.03)	−0.03 (−0.08,0.01)	−0.03 (−0.08,0.02)	0.306

*p-trend was estimated by including the median value of each quintile as a continuous variable.

Values are β-coefficients (95% confidence intervals).

Model 1 was adjusted for age, sex, years of education, income level, social class, smoking status, alcohol consumption (units/week), test site, PAEE, clinical history (self-reported hypertension or use of anti-hypertensive medication or self-reported hyperlipidaemia or use of lipid lowering medication), weight-loss diet, fibre (NSP) intake, plasma vitamin C measurement, non-sugar containing beverage intake (tea, coffee, artificially sweetened beverages) and low-nutrient energy-dense food intake (buns, cakes, puddings, biscuits, pastries, chocolate and non-chocolate confectionary and ice-cream).

Model 2 was additionally adjusted for BMI, total energy intake and sugars (sugars from liquids were additionally adjusted for sugars from solids and vice versa, and free sugars were additionally adjusted for non-free sugars and vice versa).

Estimates after adjustment for BMI and total energy intake without adjustment for sugars, did not differ from the most adjusted model.

Metabolic risk z score: derived by summing the z-scores of the following continuous indices: waist circumference, systolic blood pressure + diastolic blood pressure, 2 h plasma glucose, fasting insulin, inverted fasting HDL cholesterol, and triglycerides.

**Significant after correction for multiple testing, *p* ≤ 0.003.

Abbreviations: C-Reactive Protein, CRP; Glycated Haemoglobin, HbA1c; Homeostasis Model Assessment of Insulin Resistance, HOMA-IR; Non-Starch Polysaccharide, NSP; Percent Total Energy Intake, %TEI; Physical Activity Energy Expenditure, PAEE; Quintiles, Q.

**Fig. 2:**
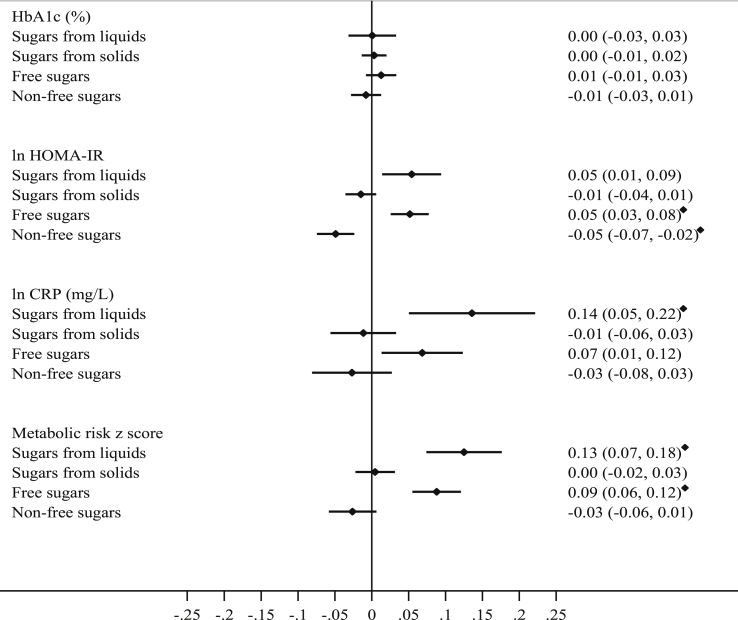
The association of intakes of sugars (per 10%TEI) and metabolic & inflammatory markers using multiple linear regression; the Fenland Study, UK (n = 9678). Values are β-coefficients (95% confidence intervals). Adjusted for age, sex, years of education, income level, social class, smoking status, alcohol consumption (units/week), test site, PAEE, clinical history (self-reported hypertension or use of anti-hypertensive medication or self-reported hyperlipidaemia or use of lipid lowering medication), weight-loss diet, fibre (NSP) intake, plasma vitamin C measurement, non-sugar containing beverage intake (tea, coffee, artificially sweetened beverages), low-nutrient energy-dense food intake (buns, cakes, puddings, biscuits, pastries, chocolate and non-chocolate confectionary and ice-cream), BMI, total energy intake and mutually adjusted for sugars (Model 2). ♦ Significant after correction for multiple testing, *p* ≤ 0.003. Metabolic risk z score: derived by summing the z-scores of the following continuous indices: waist circumference, systolic blood pressure + diastolic blood pressure, 2 h plasma glucose, fasting insulin, inverted fasting HDL cholesterol, and triglycerides; Abbreviations: C-Reactive Protein, CRP; Glycated Haemoglobin, HbA1c; Homeostasis Model Assessment of Insulin Resistance, HOMA-IR; Non-Starch Polysaccharides, NSP; Percent total energy intake, %TEI; Physical Activity Energy Expenditure, PAEE.

Associations using continuous measures (per 10%TEI) were similar to those using categorical measures and are displayed in Fig. 2 (additional detail in Supplementary Table 2).

Estimates were also largely similar when substitution with total fat, total protein and, polyols, oligosaccharides and starch was specified (Supplementary Table 3). This was with the exception of the positive association of sugars from liquids and ln HOMA-IR which was significant only if replacing protein [β-coefficient (95%CI), 0.07 (0.03,0.12)] and the positive association of sugars from liquids and ln CRP which was non-significant only if replacing protein [β-coefficient (95%CI), 0.09 (−0.01,0.19)].

### 3.4 Sensitivity and interaction analyses

Using different methods to adjust for energy intake did not substantially affect the interpretation of the associations (Supplementary Table 4). In other sensitivity analyses (Supplementary Table 5), including total sugars from milk (previously un-categorised) as sugars from liquids or restricting analyses of HbA_1c_ to participants with levels <6.5%, did not change the direction, magnitude or significance of the associations. Excluding sugar added to tea, coffee, cereal from sugars from solids, shifted the null association of sugars from solids (per 10%TEI) and all outcomes towards the inverse, making the association with ln HOMA-IR significant [β-coefficient (95%CI), −0.04 (−0.06, −0.02)]. As sugar added to tea, coffee, cereal could not be further differentiated, a conservative a priori decision was made to include it as sugars from solids. However, intakes of tea and coffee were higher than of cereal in the Fenland Study (data not shown), therefore sugar added to tea or coffee is likely to have contributed the larger proportion. Including sugar added to tea, coffee, cereal in sugars from liquids, strengthened the significant positive association of sugars from liquids (per 10%TEI) and all outcomes, including the association of intake of sugars from liquids and HbA_1c_ which became significant only after this re-categorisation [0.03 (0.01,0.05)].

No significant interactions were evident for any of the sugar exposures with age (p ≥ 0.080), sex (p ≥ 0.114) or smoking status (p ≥ 0.071). Interaction terms of BMI with liquid, solid and non-free sugar were significant for HbA_1c_, ln HOMA-IR and the metabolic risk z-score (Supplementary Table 6a) and of PAEE with solid and non-free sugars for HbA_1c_ (Supplementary Table 6b). Stratified analyses by BMI status (normal weight, overweight, obese) or PAEE (below/above median) was largely consistent with one exception.; the positive association of sugars from liquids and ln HOMA-IR was stronger in obese participants [β-coefficient (95%CI), per 10%TEI: 0.14 (0.04,0.40)] than those who were of normal weight or overweight [0.04 (−0.03,0.10)]. This did not change the overall interpretation of findings.

### 3.5 Individual components of the metabolic risk z-score

Intakes of sugars from liquids and of free sugars (per 10%TEI) were significantly (after correction for multiple testing, p ≤ 0.0017) associated with higher fasting insulin, lower HDL cholesterol and higher triglycerides (Table 3). Intakes of sugars from solids and non-free sugars were associated with lower HDL cholesterol [per 10%TEI, Model 2: β-coefficient (95%CI), −0.04 (−0.05, −0.03) and −0.03 (−0.05, −0.01) respectively]. Intake of non-free sugars was associated with lower 2-h plasma glucose [−0.13 (−0.20, −0.05)] and fasting insulin [−0.05 (−0.07, −0.02)].

**Table 3:** The association of intakes of sugars from liquids, sugars from solids, free sugars and non-free sugars (%TEI) and individual components of the metabolic risk z score using multiple linear regression; The Fenland Study, UK (n = 9768).

	Sugars from liquids	Sugars from solids	Free sugars	Non-free sugars
Mean (range), %TEI	2.9 (0–36.0)	20.6 (2.4–56.5)	12.0 (0.5–46.4)	11.5 (0.6–54.6)
	per 10%TEI			
Waist circumference (cm)	0.28 (−0.15,0.71)	−0.21 (−0.44,0.01)	0.15 (−0.13,0.43)	−0.32 (−0.60,−0.05)
Systolic blood pressure (mmHg)	1.81 (0.73,2.89)*	−0.07 (−0.64,0.49)	0.43 (−0.27,1.12)	0.22 (−0.47,0.90)
Diastolic blood pressure (mmHg)	1.29 (0.57,2.02)*	−0.28 (−0.66,0.10)	0.31 (−0.15,0.77)	−0.18 (−0.63,0.28)
2 h plasma glucose (mmol/l)	0.16 (0.04,0.28)	−0.08 (−0.15,−0.02)	0.07 (−0.01,0.15)	−0.13 (−0.20,−0.05)*
ln Fasting insulin (ρmol/l)	0.04 (0.02,0.08)*	−0.01 (−0.03,0.01)	0.05 (0.02,0.07)*	−0.05 (−0.07,−0.02)*
HDL cholesterol (mmol/l)	−0.04 (−0.07,−0.02)*	−0.04 (−0.05,−0.03)*	−0.05 (−0.06,−0.03)*	−0.03 (−0.05,−0.01)*
ln Triglycerides (mmol/l)	0.07 (0.01,0.13)*	0.01 (−0.02,0.02)	0.06 (0.04,0.09)*	−0.02 (−0.05,0.00)

Values are β-coefficients (95% confidence intervals).

Adjusted for age, sex, years of education, income level, social class, smoking status, alcohol consumption (units/week), test site, PAEE, clinical history (self-reported hypertension or use of anti-hypertensive medication or self-reported hyperlipidaemia or use of lipid lowering medication), weight-loss diet, fibre (NSP) intake, plasma vitamin C measurement, non-sugar containing beverage intake (tea, coffee, artificially sweetened beverages), low-nutrient energy-dense food intake (buns, cakes, puddings, biscuits, pastries, chocolate and non-chocolate confectionary and ice-cream), BMI, total energy intake and mutually adjusted for sugars (Model 2).

Metabolic risk z score: derived by summing the z-scores of the following continuous indices: waist circumference, systolic blood pressure + diastolic blood pressure, 2 h plasma glucose, fasting insulin, inverted fasting HDL cholesterol, and triglycerides.

*Significant after correction for multiple testing, *p* ≤ 0.0017.

Abbreviations: C-Reactive Protein, CRP; Glycated Haemoglobin, HbA1c; Homeostasis Model Assessment of Insulin Resistance, HOMA-IR; Non-Starch Polysaccharide, NSP; Percent Total Energy Intake, %TEI; Physical Activity Energy Expenditure, PAEE.

### 3.6 Food and beverage contributors

Different foods and beverages contributed to intakes of each of the sugar intake exposures (Table 4). Sugars (per 10%TEI) in fruit, vegetables, dairy products and egg dishes, cakes, biscuits and confectionary, and fruit squash/juice drinks were not significantly (after correcting for multiple testing, p ≤ 0.003) associated with any outcome in analyses adjusted for potential confounders, including BMI, energy intake, dietary quality and mutually adjusted for sugar from all other sources (Model 2, Table 4). Sugar added to tea, coffee, cereal was significantly positively associated with all outcomes. Sugars (per 10%TEI) in 100% fruit juice [0.16 (0.06,0.25)] and other non-alcoholic beverages [0.13 (0.03,0.23)] were positively associated with the metabolic risk z-score.

**Table 4:** The association of intakes of each of sugars from liquids, sugars from solids, free sugars and non-free sugars (%TEI) from major food and beverage contributors^∗^ and metabolic & inflammatory markers using multiple linear regression; The Fenland Study, UK (n = 9678).

	Contribution to intake of sugars (g/d)	Mean (range), %TEI	n consumers^∗∗^	HbA1c (%)	ln HOMA-IR	ln CRP (mg/L)	Metabolic risk z score
				per 10%TEI			
Sugars from liquids		2.9 (0–36.0)		0.00 (–0.03,0.03)	0.05 (0.01,0.09)	0.14 (0.05,0.22)^∗∗∗^	0.13 (0.07,0.18)^∗∗∗^
Fruit squash/juice drink	33%	0.9 (0–22.0)	5135	0.03 (−0.02,0.08)	0.05 (–0.01,0.11)	0.12 (–0.00,0.25)	0.07 (–0.01,0.14)
Fruit juice (100%)^a^	33%	1.1 (0–16.4)	7922	−0.04 (−0.10,0.02)	0.06 (−0.01,0.14)	0.10 (−0.06,0.26)	0.16 (0.06,0.25)^∗∗∗^
Non-alcoholic beverages	20%	0.6 (0–23.9)	7172	0.00 (−0.06,0.07)	0.00 (−0.08,0.08)	0.11 (−0.05,0.28)	0.13 (0.03,0.23)^∗∗∗^
Alcoholic beverages^b^	13%	0.5 (0–4.8)	7900	−0.34 (−0.55,−0.14)^∗∗∗^	−0.54 (−0.79,−0.29)^∗∗∗^	0.54 (−0.01,1.08)	−0.42 (−0.75,−0.10)
Sugars from solids		20.0 (2.2–56.5)		0.00 (−0.01,0.02)	−0.01 (−0.04,0.01)	−0.01 (−0.06,0.03)	0.00 (−0.02,0.03)
Cakes, biscuits & confectionary	29%	5.3 (0–44.9)	9625	0.01 (−0.03,0.04)	0.01 (−0.04,0.05)	−0.13 (−0.22,−0.04)	0.00 (−0.05,0.06)
Fruit	29%	5.9 (0–52.1)	9542	−0.00 (−0.03,0.02)	−0.04 (−0.07,−0.01)	−0.05 (−0.11,0.01)	−0.03 (−0.06,0.01)
Dairy products & egg dishes	21%	4.3 (0–20.0)	9655	−0.00 (−0.04,0.03)	−0.04 (−0.09,0.00)	0.03 (−0.07,0.13)	−0.02 (−0.08,0.04)
Sugar added to tea, coffee, cereal	15%	1.2 (0–15.1)	3668	0.06 (0.02,0.10)^∗∗∗^	0.12 (0.07,0.17)^∗∗∗^	0.25 (0.14,0.36)^∗∗∗^	0.18 (0.12,0.25)^∗∗∗^
Free sugars		11.8 (0.4–46.4)		0.01 (−0.01,0.03)	0.05 (0.03,0.08)^∗∗∗^	0.07 (0.01,0.12)	0.09 (0.06,0.12)^∗∗∗^
Cakes, biscuits & confectionary	44%	5.1 (0–44.8)	9625	0.03 (−0.02,0.08)	0.05 (−0.01,0.11)	0.12 (−0.00,0.25)	0.07 (−0.00,0.15)
Sugar added to tea, coffee, cereal	25%	1.2 (0–15.1)	3668	0.06 (0.02,0.10)^∗∗∗^	0.12 (0.07,0.17)^∗∗∗^	0.25 (0.14,0.36)^∗∗∗^	0.18 (0.12,0.25)^∗∗∗^
Fruit squash/juice drink	13%	0.9 (0–22.0)	5135	0.03 (−0.02,0.08)	0.05 (−0.01,0.11)	0.12 (−0.00,0.25)	0.07 (−0.01,0.14)
Fruit juice (100%)^a^	10%	1.1 (0–16.4)	7922	−0.04 (−0.10,0.02)	0.06 (−0.01,0.14)	0.10 (−0.06,0.26)	0.16 (0.06,0.25)^∗∗∗^
Non-free sugars		11.7 (0.6–54.6)		−0.01 (−0.03,0.01)	−0.05 (−0.07,−0.02)^∗∗∗^	−0.03 (−0.08,0.03)	−0.03 (−0.06,0.01)
Fruit	50%	5.8 (0–52.1)	9542	−0.00 (−0.03,0.02)	−0.04 (−0.07,−0.01)	−0.05 (−0.11,0.01)	−0.03 (−0.06,0.01)
Dairy products & egg dishes	28%	3.3 (0–16.8)	9655	−0.00 (−0.05,0.04)	−0.06 (−0.12,−0.00)	0.02 (−0.11,0.14)	−0.01 (−0.08,0.06)
Vegetables	11%	1.3 (0–9.5)	9667	−0.01 (−0.16,0.15)	0.20 (0.01,0.39)	0.40 (−0.00,0.81)	0.12 (−0.12,0.36)

^∗^Foods or beverages that contributed greater than 10% to each sugar intake (g/d) exposure.

^∗∗^Non-consumers included as 0%TEI.

Adjusted for age, sex, years of education, income level, social class, smoking status, alcohol consumption (units/week), test site, PAEE, clinical history (self-reported hypertension or use of anti-hypertensive medication or self-reported hyperlipidaemia or use of lipid lowering medication), weight-loss diet, fibre (NSP) intake, plasma vitamin C measurement, non-sugar containing beverage intake (tea, coffee, artificially sweetened beverages), low-nutrient energy-dense food intake (buns, cakes, puddings, biscuits, pastries, chocolate and non-chocolate confectionary and ice-cream), BMI, total energy intake and mutually adjusted for sugars from all other sources (Model 2).

^∗∗∗^Significant after correction for multiple testing, *p* ≤ 0.003.

Metabolic risk z score: derived by summing the z-scores of the following continuous indices: waist circumference, systolic blood pressure + diastolic blood pressure, 2 h plasma glucose, fasting insulin, inverted fasting HDL cholesterol, and triglycerides.

Abbreviations: C-Reactive Protein, CRP; Glycated Haemoglobin, HbA1c; Homeostasis Model Assessment of Insulin Resistance, HOMA-IR; Non-Starch Polysaccharide, NSP; Percent Total Energy Intake, %TEI; Physical Activity Energy Expenditure, PAEE.

a Not adjusted for plasma vitamin C.

b Adjustment for a categorical alcohol consumption (quintiles) variable in place of continuous variable (units/week) due to collinearity.

Sugars from alcoholic beverages contributed >10% of intake (g/d) of sugars from liquids. Due to collinearity with alcohol consumption (units/week) in this dataset, we were unable to examine the association with metabolic and inflammatory markers.

### 3.7 Other analyses

Participants classified as obese reported consuming less sugars from solids, non-free sugars, fruit, fruit juice, and alcoholic beverages, and more sugars from liquids, dairy products and egg dishes, fruit squash/juice drinks and non-alcoholic beverages than normal weight individuals (Supplementary Table 7). There was no significant trend in consumption of free sugars or of sugar added to tea, coffee, cereal across BMI categories.

## 4 Discussion

### 4.1 Summary of findings

Intakes of sugars from liquids and of free sugars were adversely associated with glycaemia and inflammatory makers. In contrast, intakes of sugars from solids and non-free sugars were either inversely, or not, associated with individual markers. Associations were similar whether sugars were replaced with fat; protein; polyols, oligosaccharides and starch; or a combination of macronutrients. Examining the major food and beverage contributors to sugar intakes indicated that the positive associations reported for free sugars were explained by the contribution of beverages to free sugar intake. Moreover, sugars from foods were not associated with glycaemia and inflammatory markers irrespective of whether they were free or non-free sugars.

### 4.2 Current findings in context

There are few data with which to compare our results. In keeping with our findings of a positive association of sugars from liquids and adverse metabolic markers, a study of American adults reported a positive association of sugar intake from beverages and mortality risk along with an inverse association of sugars from foods [20]. A second study of Canadian youth at risk of obesity reported an association of added sugars from liquid sources but not solid sources with higher fasting glucose and insulin levels [21].

Our findings are in line with the substantial evidence for sugar-sweetened beverage intake and adverse metabolic outcomes [8], [22], [23], [24], [25], [26], [27]. Previous studies have reported different associations for different sweet beverages [9], [28] including both positive [29], [30] and null associations [31], [32] for fruit juice intake. We observed significant positive associations for sugars from fruit juice and other non-alcoholic beverages, and a non-significant trend toward positive associations for sugars from fruit squash/juice drinks, which could possibly be explained by there being fewer consumers of fruit squash/juice drinks (n = 5135) than fruit juice consumers (n = 7922). The consistent findings for all non-alcoholic beverages in this study may be because we examined only the sugar intake from foods and beverages. This prevents errors arising from grouping beverages with different amounts of sugar per serving and ensures that beverage exposures are comparable e.g. for portion size.

Estimated intake of free sugars was positively associated with HOMA-IR and metabolic risk; however the associations were limited to free sugars from 100% fruit juice and other non-alcoholic beverages. Restriction of intakes of free sugars is advocated in dietary guidelines [1], [5] as they are usually contained in energy dense nutrient poor foods and are associated with obesity through their contribution to energy intake. Our findings are consistent with these recommendations, but they specifically support a focus on sugars from liquids in terms of preventing potential adverse metabolic risk.

Milk sugars were not comprehensively evaluated in these analyses as we were unable to discern whether milk was consumed as a beverage or as part of a food. In sensitivity analyses we included total sugars from milk as sugars from liquids and highlighted the possibility of an inverse association with HOMA-IR, CRP and the metabolic risk. Given these results and the inverse associations reported for certain dairy product intake and metabolic diseases including type 2 diabetes [33], [34], [35], this association warrants further investigation.

### 4.3 Mechanisms

The biological mechanisms by which sugars consumed from beverages may adversely affect metabolic and inflammatory markers are unknown. Given that there are no chemical differences between these sugars and sugars found in foods, we hypothesise that the adverse metabolic profile with sugars from liquids may relate to the large quantity of rapidly absorbable sugars that can be consumed in a relatively short period of time, similar to the hypothesis previously proposed for sugar-sweetened beverages and adverse metabolic outcomes [24]. A second hypothesis is that mono- and di-saccharides (other than lactose) may have adverse metabolic effects but the effect of sugar in food is offset by the co-assimilation of other nutrients and compounds e.g. fibre. Our analyses provides some evidence to support this hypothesis as fibre intakes were significantly higher (+5 g/d) in the highest quintile of sugar from solids and non-free sugars intake compared with the lowest.

### 4.4 Strengths and limitations

The Fenland Study was designed to be representative of the Cambridgeshire region, and population characteristics are congruent with the region's demographic characteristics. However, the sample is somewhat healthier than the England average, in particular for obesity and healthy eating [36]. The Fenland Study is a well-phenotyped study with a wide range of socio-demographic, dietary, physical activity, general health, anthropometric, metabolic and inflammatory marker data available including objectively measured physical activity and plasma vitamin C. Although we cannot rule out residual confounding, the availability of detailed data enable the comprehensive adjustment for confounders and to account for biases from participants reporting non-typical diets e.g. those with hypertension and hyperlipidaemia (post-diagnosis changes) and those who reported being on a weight-loss diet. Furthermore, there was no indication of selection bias in these analyses as population characteristics of participants included and excluded from the analyses were broadly similar.

Due to the cross-sectional study design we cannot determine the direction of the associations. It is possible that participants classified as obese (and therefore at higher risk of metabolic risk) preferentially consume more sugary foods. In this study, no clear trend emerged to suggest obese participants report consuming more sugary foods leading us to hypothesise that reverse causation is not a likely explanation for our findings. However, we cannot rule out the possibility that there may be possible selective under-reporting of sugary food intake among obese participants, and if so, it would likely have attenuated the results towards the null resulting in an underestimation of the associations.

A benefit of using FFQ-derived intake data for these analyses is that FFQs rank individuals well according to habitual intake. However, as the FFQ is semi-quantitative our estimates of intake should be interpreted relative to each other and not as absolute intake values. Additionally, some misclassification might occur as estimates by FFQ were imperfect (e.g. *r* = 0.51 for total sugars, compared to estimates by 16-day food records) [12] and as exposures of different types of sugars were estimated post-hoc. Although our approach was comprehensive and the most detailed to date to our knowledge, some foods are inherently difficult to classify for example fruit juice in unsweetened tinned fruit.

## 5 Conclusion

In a large population-based cohort, higher intakes of sugars from non-alcoholic beverages and sugar added to tea, coffee, or cereal were associated with glycaemia and inflammatory markers, while sugars from foods, regardless of whether they were free or non-free sugars, were not associated. The positive associations reported here for free sugars were largely explained by the contribution of beverages to free sugar intake. This raises the possibility that adverse metabolic risk associated with sugar intake, independent of contribution to caloric intake, may be attributable to sugar intake from beverages. The current findings should stimulate the identification of biological mechanisms and also help to further inform dietary public health messages on sugars intake.

## Funding sources

The Fenland Study is funded by the Wellcome Trust and the Medical Research Council. Support from Medical Research Council programmes MC_UU_12015/1 and MC_UU_12015/5 is acknowledged.

## Statements of authorships

LO'C performed the statistical analyses, drafted the manuscript, had full access to the data, and takes responsibility for the accuracy of the analyses and the integrity of the work as a whole. FI provided input on data analyses and gave critical input to the content of the manuscript. NGF contributed to the research question, provided input on data analyses and interpretation and is the guarantor of the work. SB, SJG and NGF are principal investigators and NJW the chief principal investigator of the Fenland Study and they are responsible for study co-ordination and data collection and they gave critical input to the content of the manuscript. All authors approved the final version.

## Conflicts of interest statement

The authors declare that there are no conflicts of interest.

## Figures and Tables

**Figure:**