# Supplementary material for: Intakes and sources of dietary sugars and their association with metabolic and inflammatory markers
Source: Clin Nutr. 2018 Aug;37(4):1313–22. doi: 10.1016/j.clnu.2017.05.030 (PMC5999353; doi:10.1016/j.clnu.2017.05.030)
Supplement: Supplementary file 1 [file mmc1.pdf]

Supplementary text

Anthropometric, metabolic and inflammatory measurements

Height and weight were measured while participants were barefoot and wearing light clothing. Weight was measured using a calibrated electronic scale (TANITA model BC-418 MA; Tanita, Tokyo, Japan). Height was measured with a wall mounted stadiometer (SECA 240; Seca, Birmingham, UK). BMI was calculated as weight (kg) divided by height squared (m^2). Waist circumference was measured midway between the lower rib margin and the iliac crest using a non-stretchable fibre-glass insertion tape (D loop tape; Chasmors Ltd, London, UK). Blood pressure was measured in a sitting position using an Omcron M4-1 automatic blood pressure monitor (Omron Healthcare Inc, UK). Measurements were taken three times at 1-3 minute intervals and the mean used for analyses.

Procedures for measurement of blood analytes

Fasting venous blood samples were taken and were followed by a standard 75g oral glucose tolerance test with further samples taken at 120 minutes. Blood samples were placed on ice, centrifuged and stored at -70°C. Plasma glucose was measured using the hexokinase method with a lower limit of detection of 0.5 mmol/L and interassay CV of 1.8% Plasma triglyceride and HDL cholesterol were measured using standard enzymatic methods; interassay CVs were 4.8% and 3.9% respectively. Insulin was determined using a two-step time resolved flourometric assay with a lower limit of detection of 1.3 pmol/L. Glycated haemoglobin (Hb_{A1c}) was measured by high-performance liquid chromatography. Interassay CVs were 1.1% at Hb_{A1c} level 5.7% (39 mmol/mol) and 1.1% at Hb_{A1c} level 9.5% (80 mmol/mol).

CRP was measured by the Core Biochemistry Assay Laboratory (CBAL), Cambridge, UK, from blood taken into plasma heparin tubes and stored at -80°C upon collection.

Plasma vitamin C was measured from blood samples taken into heparin tubes and centrifuged (10 minutes) within 30 minutes of collection. Plasma was aliquoted into cryovials with a standardised volume of metaphosphoric acid (10%) and stored at -80°C. Plasma vitamin C concentration was measured by fluorometric assay within 2 months, with a lower limit of detection of 10.0 µmol/L. Between batch imprecision was 7.9% at 27.1 µmol/L and 5.0% at 89.7 µmol/L.
